# Factors Influencing the Use of Personal Protective Equipment in the Handling of Chemotherapy Drugs Among Nursing Staff in Cancer Hospitals: A Cross‐Sectional Study

**DOI:** 10.1155/jonm/9110778

**Published:** 2026-09-07

**Authors:** Yang Zheng, Hongyan Yang, Jie Zhao, Baocui Zhang, Jingjing Luo, Haiyan Wen, Chengcheng Sheng, Qian Lu

**Affiliations:** ^1^ Department of Radiology, Peking University First Hospital, Beijing, China, pkufh.com; ^2^ Department of Nursing, Peking University First Hospital, Beijing, China, pkufh.com; ^3^ Outpatient Chemotherapy Center, Peking University First Hospital, Beijing, China, pkufh.com; ^4^ School of Nursing, Peking University, Beijing, China, pku.edu.cn

**Keywords:** cancer hospitals, chemotherapy drugs, nursing staff, personal protective equipment, PLS-SEM

## Abstract

**Aim:**

To assess the current use of personal protective equipment (PPE) among nurses handling chemotherapy drugs in cancer hospitals in China and to explore the factors associated with PPE use by nurses.

**Background:**

Nurses, the primary handlers of chemotherapy drugs, face significant exposure risks. Accurately assessing PPE use and its determinants is therefore crucial for developing targeted interventions to mitigate exposure and increase occupational safety.

**Methods:**

Convenience sampling was used to select nurses from cancer hospitals in 30 provinces in China from December 1, 2024, to January 6, 2025. A cross‐sectional survey was conducted that utilised a general information questionnaire and the Hazardous Drug Handling Questionnaire. Data analysis was performed using SPSS 24.0 and SmartPLS 4.0 software.

**Results:**

SmartPLS analysis revealed significant direct effects of interpersonal influences, workplace safety, self‐efficacy, perceived barriers, and perceived risks on PPE usage. The mediation analysis revealed that self‐efficacy, perceived risk, perceived barriers, and chemotherapy exposure knowledge mediated the relationship between chemotherapy handling experience and PPE use, whereas perceived barriers and interpersonal influences mediated the effect of workplace safety factors on PPE use. Perceived conflict of interest sharing negatively moderated the interaction between interpersonal influences and PPE usage and moderated the mediating effect of interpersonal influences.

**Conclusion:**

The results validated and extended the applicability of the health promotion model in the field of occupational safety behaviour. The findings revealed that a multilevel intervention system should be developed to increase the PPE utilisation rate among oncology nursing staff.

**Implications for Nursing Management:**

Management should foster safe environments that enable positive peer interactions, systematically transform nurses’ experience into knowledge and risk awareness, and reduce perceived nurse–patient conflicts to strengthen trust and safety norm effectiveness.

## 1. Introduction

The incidence of malignant tumours is increasing worldwide [1]. According to GLOBOCAN 2022 estimates, China accounts for 24.2% of new cancer diagnoses globally (approximately 4.82 million cases) [2, 3]. Chemotherapy is a key treatment for malignant tumours [4]. A population‐based study projected that China will have the world’s highest demand for chemotherapy, with an estimated 4.2 million new patients requiring treatment in 2040, accounting for 27.8% of global chemotherapy needs [5].

Chemotherapy drugs are hazardous drugs that not only kill or inhibit the growth of tumour cells but also cause damage to normal human tissues and organs [6, 7]. Studies have confirmed that chemotherapy drugs can invade the human body through inhalation via the respiratory tract, through intake via the digestive tract, and through contact with skin and mucous membranes, among other routes. These drugs can cause acute and chronic toxic reactions such as alopecia, fatigue, oral ulcers, bone marrow suppression, teratogenesis, carcinogenesis, and mutagenesis in the case of long‐term contact [8–10]. Nurses in cancer hospitals are directly involved in chemotherapy drug preparation, drug administration, waste disposal and other related processes. They are frequently exposed to such drugs and therefore face a high risk of occupational exposure [11]. These drug exposures directly compromise nurses’ personal safety, potentially resulting in anaemia, infertility, or even secondary malignancies. Moreover, fear of such health consequences may induce occupational anxiety, burnout syndrome, emotional exhaustion, and increased turnover intention, ultimately affecting the quality of patient care [12, 13].

Personal protective equipment (PPE), as the clothing or equipment used to protect the body of medical staff from harm when they are exposed to chemotherapy drugs, has long been the preferred means to protect nurses performing chemotherapy drug‐related operations [14]. The standardised use of PPE can reduce occupational exposure risks for nurses [15]. However, the use of PPE by nurses is not ideal [16–19]. In clinical practice, there are often problems such as incomplete wearing and delayed replacement of PPE, which increase the occupational exposure risk to nurses and threaten their health [20–22]. Therefore, a comprehensive and accurate understanding of PPE use among nurses is critical for assessing the level of occupational protection, identifying weak links, and formulating strategies to reduce occupational exposure risk.

In this study, to systematically explore the mechanisms underlying nurses’ PPE use, the Hazardous Drug Handling Questionnaire (HDHQ) developed by Polovich and Clark [23] was used. The theoretical framework guiding the study was the Factors Predicting Use of Hazardous Drug Safe‐Handling Precautions model, which was adapted from Lusk, Ronis, and Hogan [24]—who developed a model for predicting hearing protection use among construction workers—and is grounded in Pender’s Health Promotion Model (HPM) [25]. Although the HPM was originally developed for health‐promoting behaviours, it has been successfully extended to occupational protective contexts [21]. Unlike alternative frameworks, the HPM uniquely captures individual, interpersonal, and situational factors simultaneously—all of which are critical predictors of PPE use. Moreover, the concept of competing demands in the HPM provides a theoretical foundation for understanding how perceived conflict of interest (e.g., patient care versus self‐protection) may moderate nurses’ PPE behaviour.

The objectives of this study were as follows: (1) to understand the current use of PPE in handling chemotherapy drugs among nurses in cancer hospitals in China and (2) to explore the factors related to PPE use by nurses and verify the applicability of the HPM for analysing the occupational safety behaviour of nurses in a Chinese medical context.

## 2. Methods

### 2.1. Study Design and Participants

This cross‐sectional study was conducted across 30 provincial cancer hospitals in China from December 2024 to January 2025. The convenience sampling method was adopted. A total of 1386 nurses were included in the analysis. The inclusion criteria were as follows: (1) the participants were registered nurses; (2) the participants handled chemotherapy drugs as part of their job; and (3) the participants provided informed consent and participated voluntarily.

### 2.2. Instruments

#### 2.2.1. General Information Questionnaire

Through a literature review, we designed a questionnaire to collect general information about the participants, including sex, age, educational level, professional title, and marital status, on the basis of the research content and goals.

#### 2.2.2. HDHQ

The HDHQ, which was developed by Polovich and Clark [23], was used to evaluate chemotherapy handling practices. Formal permission to translate and culturally adapt the questionnaire for use in the Chinese context was obtained from the original author prior to the translation process. The first part of the questionnaire is used to assess the frequency of PPE use in four situations: hazardous drug preparation, administration, chemical waste disposal, and excreta handling. The second part of the questionnaire consists of seven subscales (predictors), including chemotherapy exposure knowledge (12 items), self‐efficacy for using PPE (7 items), perceived barriers to using PPE (13 items), perceived risks of chemotherapy exposure (3 items), workplace safety climate (21 items), perceived conflicts of interest (6 items; defined as the nurse’s perception that her or his own safety practices conflict with patients’ expectations for immediate care), and interpersonal influences (7 items). The instrument was found to be valid and reliable when used by oncology nurses in a study by Polovich and Clark [23], with the Cronbach’s *α* values for each scale ranging from 0.70 to 0.93. The research team translated the scale into Chinese. Following the Brislin translation model, the HDHQ was translated, back‐translated, culturally adapted, and pre‐tested to form the Chinese version, and its reliability and validity were tested among 406 nurses in Beijing. The Chinese version of the HDHQ has been published [26]. The Cronbach’s *α* ranged from 0.742 to 0.971.

### 2.3. Data Collection

Before the survey was administered, the researchers contacted managers at cancer hospitals in 30 provinces (autonomous regions and municipalities) in China to explain the purpose and significance of the survey. After approval was obtained from the hospital’s nursing department, Questionnaire Star software was used to distribute the electronic questionnaires. Participants who met the inclusion criteria responded anonymously and voluntarily. To prevent duplicate submissions, survey entries were restricted to one entry for each IP address, computer, and mobile device. The questionnaire could be submitted only when all the required components were fully completed. A total of 1800 nurses were invited, and 1684 responded. After 298 invalid cases were excluded, 1386 valid responses were retained, yielding a final response rate of 77.0%.

### 2.4. Data Analysis

The data were analysed using SPSS version 24 and SmartPLS 4.0. Continuous variables with a normal distribution are presented as the mean ± standard deviation, whereas categorical variables are presented as the frequency and composition ratio. In cases where the data were not normally distributed, medians and interquartile ranges are presented. To explore the complex interactions between observable and latent variables in the dataset, structural equation modelling using partial least squares (PLS‐SEM), a statistical approach that combines factor analysis and regression, was employed. This study involved the development of a measurement model, assessments of its validity and consistency, and the development of a path model to examine the associations among unobserved variables. Compared with traditional SEM approaches, the PLS‐SEM method has advantages because it can handle small sample sizes and nonnormal data distributions [27].

### 2.5. Ethical Considerations

This study was approved by the Biomedical Ethics Committee of Peking University. The ethics approval number of the study is (IRB00001052‐24011). The survey was voluntary, anonymous, and confidential. Subjects could enrol in the study once they had read the explanations (including the study objectives, participants’ anonymity, and the possibility of participating in the study) on the first page of the online questionnaire. Participants could then confirm their agreement by clicking the ‘I Agree’ button.

## 3. Results

### 3.1. Participant Characteristics

Overall, 1386 nurses participated in the study. The details of the participants are shown in Table 1. The mean age of the participants in the study was 34.54 ± 6.30 years, ranging from 20 to 56 years, and their mean number of years of work experience involving the handling of chemotherapy drugs was 10.78 ± 6.45 years, ranging from 1 year to 34 years. Most of the nurses were female (98.6%), and the average number of assigned chemotherapy patients on each workday was 17.25 ± 26.31.

**TABLE 1:** Demographic information of the survey participants (*N* = 1386).

Category	Attribute	Number of participants	Percentage of respondents
Sex	Male	19	1.4
	Female	1367	98.6

Educational level	Diploma	5	0.4
	Associate degree	132	9.5
	Bachelor’s degree	1217	87.8
	Master’s degree or above	32	2.3

Professional title	Junior nurse	118	8.5
	Nurse practitioner	473	34.1
	Nurse in charge	730	52.7
	Associate chief nurse or above	65	4.7

Marital status	Single	353	25.5
	Married	1033	74.5

### 3.2. PPE Use During Different Tasks Involved in Handling Chemotherapy Drugs

Among the 1386 nurses, 731 (52.7%) participated in the formulation of chemotherapy drugs, 1321 (95.3%) participated in the administration of chemotherapy drugs, 948 (68.4%) participated in the handling of chemotherapy waste, and 170 (12.3%) participated in the handling of the excreta of chemotherapy patients; for the above four situations, the scores for PPE use were 2.33 (1.17, 3.67), 2.00 (1.00, 3.00), 2.00 (1.00, 3.20), and 3.20 (1.60, 5.00), respectively. The specific scores are shown in Table 2.

**TABLE 2:** Reported PPE use frequencies for individual tasks among nurses.

PPE	Case (%)					
	Always	76%–99%	51%–75%	26%–50%	1%–25%	Never
*Preparation (n = 731)*						
Mask	419 (57.3)	74 (10.1)	40 (5.5)	25 (3.4)	37 (5.1)	136 (18.6)
Double gloves	366 (50.1)	107 (14.6)	63 (8.6)	36 (4.9)	53 (7.3)	106 (14.5)
Biological Safety Cabinet	223 (30.5)	65 (8.9)	34 (4.7)	16 (2.2)	56 (7.7)	337 (46.1)
Eye protection	196 (26.8)	74 (10.1)	53 (7.3)	51 (7.0)	88 (12.0)	269 (36.8)
Gowns labelled for use with chemotherapy	145 (19.8)	71 (9.7)	37 (5.1)	28 (3.8)	59 (8.1)	391 (53.5)
Gloves labelled for use with chemotherapy	139 (19.0)	63 (8.6)	38 (5.2)	31 (4.2)	55 (7.5)	405 (55.4)

*Administration (n = 1321)*						
Mask	762 (57.7)	128 (9.7)	64 (4.8)	46 (3.5)	59 (4.5)	262 (19.8)
Double gloves	460 (34.8)	182 (13.8)	130 (9.8)	99 (7.5)	160 (12.1)	290 (22.0)
Eye protection	237 (17.9)	104 (7.9)	88 (6.7)	70 (5.3)	176 (13.3)	646 (48.9)
Gloves labelled for use with chemotherapy	187 (14.2)	107 (8.1)	84 (6.4)	58 (4.4)	107 (8.1)	778 (58.9)
Gowns labelled for use with chemotherapy	162 (12.3)	91 (6.9)	68 (5.1)	47 (3.6)	107 (8.1)	846 (64.0)

*Disposal (n = 948)*						
Mask	555 (58.5)	85 (9.0)	44 (4.6)	23 (2.4)	44 (4.6)	197 (20.8)
Double gloves	383 (40.4)	100 (10.5)	73 (7.7)	59 (6.2)	95 (10.0)	238 (25.1)
Eye protection	185 (19.5)	66 (7.0)	44 (4.6)	60 (6.3)	114 (12.0)	479 (50.5)
Gloves labelled for use with chemotherapy	182 (19.2)	65 (6.9)	39 (4.1)	34 (3.6)	79 (8.3)	549 (57.9)
Gowns labelled for use with chemotherapy	152 (16.0)	64 (6.8)	32 (3.4)	45 (4.7)	68 (7.2)	587 (61.9)

*Handling excreta (n = 170)*						
Mask	100 (58.8)	23 (13.5)	10 (5.9)	6 (3.5)	10 (5.9)	21 (12.4)
Double gloves	90 (52.9)	28 (16.5)	10 (5.9)	10 (5.9)	12 (7.1)	20 (11.8)
Eye protection	60 (35.3)	20 (11.8)	15 (8.8)	8 (4.7)	19 (11.2)	48 (28.2)
Gloves labelled for use with chemotherapy	59 (34.7)	21 (12.4)	12 (7.1)	8 (4.7)	13 (7.6)	57 (33.5)
Gowns labelled for use with chemotherapy	54 (31.8)	20 (11.8)	11 (6.5)	11 (6.5)	16 (9.4)	58 (34.1)

### 3.3. PLS‐SEM

#### 3.3.1. Analysis of the Reflective Measurement Model

The outer loading scores for each retained item are shown in Table 3. The outer loading scores ranged from 0.510 to 0.936, with no indicator loading score below 0.4 [28]. Item SE5 was removed from the self‐efficacy subscale due to its low outer loading score (0.323). Statistical indicators obtained from SmartPLS 4.0 software revealed that the Cronbach’s alpha values ranged between 0.896 and 0.970 for all the latent variables. The composite reliability (CR) ranged between 0.934 and 0.973 for all the latent variables. A CR value greater than 0.70 is recommended as the acceptance criterion [27].

**TABLE 3:** Construct reliability and validity.

Construct	Item	Loading	Cronbach *α*	CR	AVE
Self‐efficacy	SE1	0.761	0.931	0.945	0.743
	SE2	0.861			
	SE3	0.886			
	SE4	0.898			
	SE6	0.873			
	SE7	0.886			

Perceived barriers	PB1	0.510	0.926	0.934	0.525
	PB2	0.518			
	PB3	0.733			
	PB4	0.639			
	PB5	0.656			
	PB6	0.727			
	PB7	0.783			
	PB8	0.842			
	PB9	0.813			
	PB10	0.826			
	PB11	0.791			
	PB12	0.807			
	PB13	0.674			

Perceived risks	PR1	0.887	0.896	0.935	0.827
	PR2	0.936			
	PR3	0.906			

Perceived conflict of interest	PCOI1	0.817	0.938	0.951	0.764
	PCOI2	0.854			
	PCOI3	0.892			
	PCOI4	0.893			
	PCOI5	0.884			
	PCOI6	0.901			

Workplace safety climate	WSC1	0.612	0.970	0.973	0.634
	WSC2	0.681			
	WSC3	0.759			
	WSC4	0.842			
	WSC5	0.863			
	WSC6	0.884			
	WSC7	0.814			
	WSC8	0.806			
	WSC9	0.531			
	WSC10	0.803			
	WSC11	0.857			
	WSC12	0.876			
	WSC13	0.877			
	WSC14	0.868			
	WSC15	0.803			
	WSC16	0.809			
	WSC17	0.733			
	WSC18	0.761			
	WSC19	0.763			
	WSC20	0.833			
	WSC21	0.850			

Convergent validity was assured by evaluating the importance of the outer loading scores and the average variance extracted (AVE). All latent variables had AVE values higher than 0.5, ranging between 0.525 and 0.827 (Table 3). An AVE value greater than 0.5 is recommended as the acceptance criterion [29]. The findings showed that convergent validity was high for all the latent variables. Discriminant validity was assessed using both the Fornell–Larcker criterion and the HTMT ratio. As shown in Table 4, the square root of the AVE values was greater than the correlation coefficients among the latent variables, satisfying the Fornell–Larcker criterion. More stringently, all the HTMT ratios were less than the conservative threshold of 0.850 (ranging from 0.024 to 0.722) [27]. These results validated the discriminant validity of the constructs.

**TABLE 4:** Fornell–Larcker criterion and HTMT results.

	Fornell–Larcker criterion									Heterotrait–Monotrait ratio (HTMT)							
	II	PCOI	CHE	PPE usage score	WSC	SE	CEK	PB	PR	II	PCOI	CHE	PPE usage score	WSC	SE	CEK	PB
Interpersonal influence score	**1**																
Perceived conflict of interest	−0.449	**0.874**								0.461							
Chemotherapy handling experience	−0.083	0.099	**1**							0.083	0.099						
PPE usage score	0.446	−0.255	−0.085	**1**						0.446	0.261	0.085					
Workplace safety climate	0.439	−0.245	−0.027	0.482	**0.797**					0.441	0.253	0.043	0.484				
Self‐efficacy	0.392	−0.235	−0.056	0.404	0.624	**0.862**				0.399	0.249	0.07	0.413	0.647			
Chemotherapy exposure knowledge score	0.024	−0.017	0.121	−0.045	0.015	−0.137	**1**			0.024	0.036	0.121	0.045	0.056	0.139		
Perceived barriers	−0.569	0.663	0.052	−0.382	−0.461	−0.421	−0.047	**0.725**		0.559	0.722	0.065	0.35	0.433	0.407	0.081	
Perceived risks	0.167	−0.292	−0.04	−0.06	−0.009	−0.034	0.177	−0.297	**0.91**	0.176	0.322	0.044	0.06	0.074	0.045	0.185	0.379

*Note:* II: interpersonal influence score. Bold values on the diagonal represent the square root of AVE.

Abbreviations: CEK, chemotherapy exposure knowledge score; CHE, chemotherapy handling experience; PB, perceived barriers; PCOI, perceived conflict of interest; PR, perceived risks; SE, self‐efficacy; WSC, workplace safety climate.

#### 3.3.2. Assessment of the Structural Model

Data bias was evaluated based on the inner variance inflation factor (VIF). If the VIF values in the inner model resulting from a full collinearity test were equal to or less than 3 [27], the model was considered free of common method bias. Our results revealed that all the VIF values ranged from 1.000 to 2.526 (Table 5); thus, common method bias is unlikely to be a major concern. The results of Harman’s single‐factor test further confirmed this finding, with the first unrotated factor explaining 29.41% of the total variance (below the 50% threshold) [30, 31]. Nevertheless, because our data were collected from a single source at a single time point, these statistical tests only mitigate, rather than eliminate, concerns about common method bias. Readers should interpret the findings with this limitation in mind.

**TABLE 5:** Inner VIF values.

Construct	Interpersonal influence score	PPE usage score	Chemotherapy exposure knowledge score	Perceived barriers	Perceived risks
Interpersonal influence score		1.764			
Perceived conflict of interest		1.876			
Chemotherapy handling experience			1		1.015
PPE usage score					
Workplace safety climate	1	1.989		1.638	
Self‐efficacy		1.759	1	1.638	
Chemotherapy exposure knowledge score					1.015
Perceived barriers		2.526			
Perceived risks		1.178			

**FIGURE 1:**
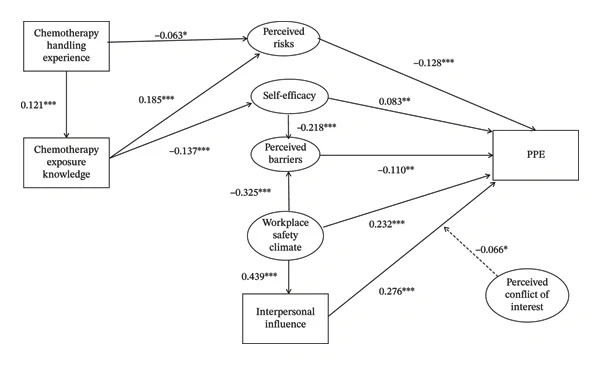
Structural model results. Standardised path coefficients (*β*) are shown next to each arrow. Solid arrows indicate direct paths; dashed arrows indicate moderating effects. Significance levels: ^∗^ *p* < 0.05; ^∗∗^ *p* < 0.01; ^∗∗∗^ *p* < 0.001.

The coefficient of determination (*R* ^2^) was used to measure the overall effect size and variance explained by the dependent variable. The model fit test results (Table 6 and Figure 1) revealed that all the exogenous constructs explained 34.2% (*R* ^2^ = 0.342) of the observed variance in the PPE use score. The prediction error was assessed using *Q* ^2^ values, with a *Q* ^2^ value greater than zero indicating the predictive relevance of the model for specific variables. For the PPE use score, the *Q* ^2^ value was 0.320. These values indicated the high predictive relevance of the model for the selected dependent variables owing to the selected independent variables. The standardized root mean square residual value was 0.068, which was less than 0.08, indicating that the model fit the data well.

**TABLE 6:** Coefficients of determination (*R* ^2^ and *Q* ^2^).

Exogenous	*R* ^2^	*Q* ^2^
PPE usage score	0.342	0.320
Self‐efficacy	0.019	0.013
Chemotherapy exposure knowledge score	0.015	0.014
Perceived risks	0.035	0.028
Interpersonal influence score	0.193	0.191
Perceived barriers	0.242	0.112

Finally, as shown in Table 7 and Figure 1, a bootstrapping method in SmartPLS 4.0 was used to determine the path coefficients and corresponding *t*‐values for both direct and mediating relationships. The SmartPLS results revealed direct relationships among interpersonal influences, workplace safety climate, self‐efficacy, perceived barriers, perceived risks, and the PPE usage score. Interpersonal influences had the most decisive influence on the PPE usage score (*β* = 0.276; *t* value = 9.72; *p* < 0.001), and workplace safety climate had the second most substantial influence on the PPE usage score (*β* = 0.232; *t* value = 6.419; *p* < 0.001). However, self‐efficacy had the lowest influence on the PPE usage score (*β* = 0.083; *t* value = 2.734; *p* < 0.01). The results of the mediating effect analysis revealed that self‐efficacy, perceived risk, perceived barriers, and chemotherapy exposure knowledge significantly mediated the relationship between chemotherapy handling experience and PPE use. Moreover, perceived barriers and interpersonal influences had mediating effects on the relationship between workplace safety climate and PPE use.

**TABLE 7:** Path coefficients.

Path	Path coefficient (*β*)	*t* Values	*p*	Boot LLCI	Boot ULCI	Decision
II ⟶ PPE usage score	0.276	9.720	< 0.001	0.220	0.331	Supported
CHE ⟶ CEK	0.121	5.069	< 0.001	0.075	0.167	Supported
CHE ⟶ PR	−0.063	2.354	0.019	−0.115	−0.011	Supported
WSC ⟶ II	0.439	17.003	< 0.001	0.386	0.489	Supported
WSC ⟶ PPE usage score	0.232	6.419	< 0.001	0.160	0.301	Supported
WSC ⟶ PB	−0.325	8.239	< 0.001	−0.404	−0.248	Supported
SE ⟶ PPE usage score	0.083	2.734	0.006	0.025	0.144	Supported
SE ⟶ PB	−0.218	5.984	< 0.001	−0.288	−0.146	Supported
CEK ⟶ SE	−0.137	5.190	< 0.001	−0.189	−0.085	Supported
CEK ⟶ PR	0.185	7.305	< 0.001	0.136	0.233	Supported
PB ⟶ PPE usage score	−0.110	2.794	0.005	−0.191	−0.037	Supported
PR ⟶ PPE usage score	−0.128	5.082	< 0.001	−0.178	−0.079	Supported
CHE ⟶ CEK ⟶ SE	−0.017	3.555	< 0.001	−0.026	−0.008	Supported
SE ⟶ PB ⟶ PPE usage score	0.024	2.485	0.013	0.008	0.045	Supported
CEK ⟶ PR ⟶ PPE usage score	−0.024	4.150	< 0.001	−0.036	−0.014	Supported
CHE ⟶ CEK ⟶ PR	0.022	4.067	< 0.001	0.013	0.034	Supported
CEK ⟶ SE ⟶ PPE usage score	−0.011	2.379	0.017	−0.022	−0.003	Supported
WSC ⟶ II ⟶ PPE usage score	0.121	8.315	< 0.001	0.093	0.150	Supported
CEK ⟶ SE ⟶ PB	0.030	3.984	< 0.001	0.017	0.045	Supported
CHE ⟶ CEK ⟶ SE ⟶ PB	0.004	3.103	0.002	0.002	0.006	Supported
CHE ⟶ CEK ⟶ SE ⟶ PPE usage score	−0.001	2.058	0.040	−0.003	0.000	Supported
CEK ⟶ SE ⟶ PB ⟶ PPE usage score	−0.003	2.248	0.025	−0.007	−0.001	Supported
CHE ⟶ CEK ⟶ SE ⟶ PB ⟶ PPE usage score	0.0004	2.007	0.045	−0.001	0.000	Supported
CHE ⟶ CEK ⟶ PR ⟶ PPE usage score	−0.003	3.139	0.002	−0.005	−0.001	Supported
CHE ⟶ PR ⟶ PPE usage score	0.008	2.194	0.028	0.001	0.016	Supported
WSC ⟶ PB ⟶ PPE usage score	0.036	2.651	0.008	0.012	0.065	Supported
PCOI × PB ⟶ PPE usage score	0.059	1.763	0.078	−0.008	0.120	Not Supported
PCOI × WSC ⟶ PPE usage score	0.005	0.143	0.886	−0.062	0.064	Not Supported
PCOI × II ⟶ PPE usage score	−0.066	1.990	0.047	−0.133	−0.003	Supported
PCOI × SE ⟶ PPE usage score	0.007	0.226	0.821	−0.049	0.072	Not Supported
PCOI × PR ⟶ PPE usage score	−0.025	1.065	0.287	−0.072	0.022	Not Supported

*Note:* II: interpersonal influence score.

Abbreviations: CEK, chemotherapy exposure knowledge score; CHE, chemotherapy handling experience; PB, perceived barriers; PCOI, perceived conflict of interest; PR, perceived risks; SE, self‐efficacy; WSC, workplace safety climate.

**FIGURE 2:**
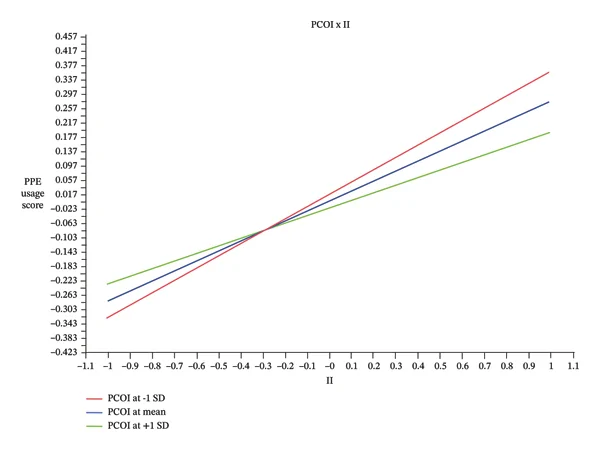
Moderating effect of PCOI on the relationship between II and PPE use. II: interpersonal influence score; PCOI: perceived conflict of interest. The three lines represent the relationship at different levels of PCOI (−1 SD, mean, +1 SD).

Although interpersonal influences had significant effects on PPE use scores, perceived conflicts of interest also played an influencing role (Figure 2). The results revealed that perceived conflicts of interest were a significant moderating variable. The results shown in Table 7 reveal that the interpersonal influence score and the perceived conflict of interest interaction term had negative effects on the PPE use score (*β* = −0.066, *t* = 1.990, *p* = 0.047). Additionally, the 95% confidence interval was (−0.133, −0.003), excluding 0. These findings indicated that perceived conflicts of interest had negative moderating effects on the impact of the interpersonal influence score on the PPE use score. The simple slope analysis of this moderating effect (Figure 2) revealed that as the perceived conflict of interest increased, the influence of interpersonal influence factors on the PPE use scores of nurses gradually decreased.

Finally, the moderating effect of perceived conflicts of interest on the ‘WSC ⟶ II ⟶ PPE usage score’ mediation model was tested (Table 8). The adjusted mediation path coefficient was −0.098, and the 95% confidence interval was (−0.139, −0.058), excluding 0. These results indicated that the moderated mediation influence was significant and that the lower the perceived conflict of interest was, the easier it was for workplace safety climate factors to influence PPE use through interpersonal influences.

**TABLE 8:** Moderated mediation test results.

	Perceived conflict of interest	Path coefficient	Standard deviation	*t* value	*p* value	95% confidence interval	
WSC ⟶ II ⟶ PPE usage score	M + 1SD	0.142	0.029	4.845	< 0.001	0.087	0.200
	M	0.304	0.037	8.200	< 0.001	0.231	0.376
	M − 1SD	0.223	0.027	8.322	< 0.001	0.172	0.276

Index of conditional mediation		−0.098	0.024	−4.033	< 0.001	−0.139	−0.058

*Note:* II: interpersonal influence score.

Abbreviation: WSC, workplace safety climate.

## 4. Discussion

### 4.1. The State of PPE Use in the Handling of Chemotherapy Drugs

PPE use differed across scenarios (such as preparation, administration, waste disposal, and patient excreta disposal). PPE was most commonly used in the disposal of the excrement of chemotherapy patients, a finding that is in sharp contrast to the results reported by Graeve et al. [32] and Callahan et al. [33], who consistently reported the lowest PPE use scores for this scenario.

These findings may be due to the strengthening of hospital and department training in Chinese cancer hospitals, which has significantly improved the understanding among nurses of the drug components in the excreta of chemotherapy patients. In addition, the traditional Chinese concept of ‘excreta pollution’ is highly sensitive; thus, PPE is more commonly used in the process of handling the excreta of patients with chemotherapy. This difference shows that cognitive and cultural factors play key roles in changing nurses′ PPE compliance behaviour. Furthermore, these results provide practical ideas for formulating protective intervention strategies that are consistent with nursing practices and integrating them at regional, social and cultural scales.

There were differences in the use of different types of PPE among nurses. In China, when nurses handled chemotherapy drugs, masks and double gloves were used the most, while the utilisation rates of specialised gloves and gowns designed for chemotherapy scenarios were the lowest, results that are not consistent with the findings of previous studies [22]. Graeve et al. [32] and Polovich and Clark [23] reported that chemotherapy gloves and protective clothing are the most commonly used approaches. This discrepancy may result from the different requirements for PPE use in various countries, which may affect the clinical practice of nurses [34]. In addition, basic PPE, such as masks and rubber gloves, is cost‐effective and easy to wear and operate; thus, these types of PPE are more suitable for use in clinical practice because of the high patient turnover rate and high workload in China. In the international medical system, protection guidelines for hazardous drugs in some countries have been restricted and supported by policies and laws and evaluated by regular inspections and audits [35], thus further promoting the use of PPE such as gloves and protective clothing in scenarios involving the handling of chemotherapy drugs. In summary, differences in the use of different types of PPE provide a basis for analysing the influence of environmental variables such as the national medical system, working mode, and resources on the protective behaviours of nurses [36]. These differences may also reflect the choices made by nurses in different countries in terms of protection focus and resource constraints given the different levels of nursing experience and nursing system pressure.

### 4.2. Factors Influencing the Use of PPE in the Context of Handling Chemotherapy Drugs

The results of this study validated the HPM and expanded the understanding of PPE use among nurses. In predicting PPE use behaviour among oncology nurses, ‘workplace safety climate’ at the organisational level and ‘chemotherapy handling experience’ at the individual level were the key factors associated with nurses’ self‐efficacy, chemotherapy exposure knowledge, interpersonal influences, and risk and barrier perception; thus, these factors may serve as important foundations for safe behaviour. In addition, ‘perceived conflicts of interest’, as a key moderating variable, influenced whether interpersonal influences could be effectively translated into the safe handling of chemotherapy drugs in practical scenarios.

Workplace safety climate is the core component representing ‘situational influence’ in the HPM. A positive and supportive environment is a key situational factor associated with PPE use among nurses [32, 37]. A workplace safety climate involves the direction, goals and processes related to safety in an organisation and represents an organisation’s commitment to safety [38]. This factor may be associated with greater PPE use and compliance among nurses [39] and also increases nurses’ PPE use by affecting barrier perception and interpersonal influences. A safe and protective environment in the workplace may support hospital managers in improving the use of protective equipment in the context of managing chemotherapy drugs and thus may improve the use of PPE among nurses. To improve nurses’ PPE use, sufficient equipment and materials must be provided, an optimised supervision and feedback mechanism must be established, processes must be streamlined to reduce nurses’ perception of barriers to PPE use [40], positive interpersonal influences (such as peer supervision, leading by example, and team norms) must be strengthened [41, 42], and a safe workplace must be established.

Additionally, chemotherapy handling experience and individual characteristics and experiences attributed to the HPM support the model’s premise that individual characteristics and experiences influence behaviours. In contrast to other studies [43, 44], this study revealed that the chemotherapy experience was not simply or directly associated with PPE use. Experience, which mainly serves as a potential cognitive resource, must be transformed into a series of internal psychological processes, such as systematic chemotherapy exposure knowledge, stable self‐efficacy, acute risk perception, and acute barrier perception, to robustly support normative behaviour. Four key mediating targets for the transformation of experience into behaviour were identified. Based on these targets, a precise starting point for PPE use intervention was identified. In particular, nurses must gain experience, and the transformation of experience into knowledge should be promoted through structured training, with the aims of strengthening self‐efficacy through successful feedback and structured interventions [41, 45], maintaining risk awareness through case education, and listening to the feedback of senior nurses through participatory management to improve the system. Notably, due to its low outer loading score (0.323), the item SE5 (‘Reuse of disposable PPE makes me feel less protected’) has been removed from the self‐efficacy subscale. This item may have captured a perception of safety or risk regarding PPE reuse rather than the core construct of self‐efficacy. Following its removal, the CR (0.945) and AVE (0.743) remained above recommended thresholds, indicating that the construct’s validity and reliability were not compromised.

The results of this study reveal a deep contradiction that is often overlooked in occupational safety research but is critically related to nursing practice: when nurses perceive that strict PPE use may conflict with the optimisation of patients’ psychological feelings or immediate care [46], the positive association between social drivers of safe behaviour and PPE use is significantly weakened. With increasing perceived conflicts of interest, this positive association between interpersonal influences and behaviour significantly weakens. This moderating effect may influence the entire mediating path of ‘workplace safety climate ⟶ interpersonal influences ⟶ safe handling precautions’. This tension is not unique to PPE compliance. A growing body of evidence indicates that oncology nurses routinely navigate multidimensional role demands that require them to balance immediate patient care responsibilities against their own professional and physical well‐being [47]. These demands—ranging from symptom assessment and advance care planning to caregiver support—are often compounded by unclear role delineation, limited communication training, and insufficient organisational support, creating a context in which competing demands such as those captured by perceived conflict of interest (PCOI) become particularly salient. At the psychological level, addressing this tension requires that the most effective safety‐related cultural factors be considered in the context of nurses’ professional identity and nurse–patient relationships [48]. By reshaping the narrative, improving communication skills and helping nurses address internal ethical conflicts, safety norms can be truly internalised, which may facilitate conflict‐free conscious actions.

Despite the moderate *R* ^2^, the significant path coefficients (self‐efficacy, interpersonal influences, perceived risks, and perceived barriers) provide clear, actionable targets for intervention. This moderate *R* ^2^ does not limit the applicability of the model for designing interventions, as the significant predictors offer direct levers for change.

A concrete example of an effective evidence‐based intervention is provided by Crickman [49], who implemented a 30‐min online education module combined with PPE standardisation and hazard identification in an inpatient oncology unit. Using a pre‐post design, they reported significant improvements in nurses’ knowledge scores (from 10.5 to 11.2 out of 12, *p* < 0.001) and correct PPE doffing (from 11% to 80%). Although the authors did not frame their intervention using our theoretical constructs, its components align with several key factors we identified: institutional standardisation supports a workplace safety climate; clear doffing protocols reduce perceived barriers; step‐by‐step guidance may enhance self‐efficacy; and real‐time coaching provides interpersonal influence. Thus, this programme offers a replicable, evidence‐based template for improving the safety of chemotherapy among nurses.

The model explained 34.2% of the variance in PPE use, leaving room for future extensions. Theoretically, additional constructs not included in the current model, such as policy enforcement, legal regulatory frameworks, the availability of specialised PPE, workload and shift characteristics, risk tolerance, safety motivation, and external regulatory inspections [50, 51], could be integrated into future research. For example, consistent policy enforcement may reinforce the effect of workplace safety climate by mandating PPE use; conversely, heavy workload may weaken nurses’ PPE adherence [43]. Future research could examine how these factors interact with the constructs identified in our model to refine intervention strategies.

### 4.3. Sampling Strategy and External Validity

In the present study, convenience sampling exclusively from provincial‐level cancer hospitals in China was employed. Compared with municipal, county‐level, and rural oncology units, provincial‐level cancer hospitals are typically tertiary referral centres equipped with better resources, stronger safety infrastructure, and more rigorous PPE training and enforcement. Consequently, our sample likely over‐represents nurses working in resource‐rich, high‐compliance environments and under‐represents those from lower resource, lower compliance settings—precisely the environments where PPE compliance may be lowest. Therefore, we propose that the true mean PPE compliance among all Chinese oncology nurses is likely lower than the level observed in our study. That is, our findings probably overestimate actual PPE use in the broader nursing population. This bias should be accounted for when interpreting the reported compliance rates and the relative importance of specific determinants (e.g., perceived barriers may play an even larger role in excluded settings). In future studies, multi‐stage stratified random sampling across different tiers of cancer care institutions (provincial, municipal, and county‐level) and across geographic regions should be adopted to improve representativeness and external validity.

### 4.4. Limitations

This study has several limitations. First, convenience sampling was used to collect data from nursing staff in oncology hospitals; thus, the findings may not be generalisable to all nurses. In the future, nationwide multicentre stratified sampling studies involving medical institutions of varying levels, types, and regions should be conducted. Second, our findings may have been affected by the self‐report measures used and thus could be vulnerable to reporting bias. In particular, social desirability bias may have inflated reported PPE use and led to an overestimation of the positive path coefficients or an underestimation of the negative path coefficients. The use of an IP‐restricted online survey may have further reduced the perceived anonymity, discouraging honest disclosure of noncompliance. In future research, additional data collection approaches incorporating multiple methods, such as face‐to‐face interviews, direct observations, and environmental monitoring, should be used. Third, we did not collect information on nurses’ reproductive history or reproductive problems. This is a notable limitation given the well‐documented reproductive toxicity of chemotherapy drugs (e.g., menstrual disorders, infertility, and spontaneous abortion). The absence of these data limits our ability to assess the full health impact of occupational exposure and whether reproductive concerns might influence PPE use behaviour (e.g., those planning pregnancy or those with prior reproductive issues might adopt stricter PPE use). Future studies should include reproductive health outcomes to address this gap. Fourth, despite a high response rate (77.0%), nonresponse bias cannot be completely ruled out because of the sensitive topic, which may have led to an overestimation of PPE use.

## 5. Conclusion

This study revealed that the use of PPE among nurses in Chinese cancer hospitals needs to improve. On the basis of Pender’s HPM, a structural equation model was used to systematically explore and verify the complex mechanisms affecting the use of PPE by nurses in Chinese cancer hospitals in the context of handling chemotherapy drugs. In future research, this model can be used to understand the core mechanisms; subsequently, PPE use can be improved through evidence‐based intervention programmes to protect the self‐interests and occupational safety of nurses. Given that the present sample was drawn exclusively from provincial (tertiary) hospitals, the generalisability of these findings to other settings may be limited.

### 5.1. Implications for Nursing Management

This study provides key management insights for the construction of a systematic occupational safety protection system in the context of handling chemotherapy drugs. The core task of nursing management should shift from scattered individual training to collaborative interventions involving organisational systems and individuals. Systematic evaluations of positive interpersonal interactions and peer supervision within safe workplace environments are needed to transform nurses′ experience into structured knowledge to recognise high‐risk behaviours and reduce barriers. Additionally, nurse–patient conflicts of interest must be resolved to ensure that management measures can be effectively transformed into safe behaviour. These recommendations are supported by the predictive relevance of the model (*Q* ^2^ = 0.320 > 0), indicating that the model can usefully inform practices despite the moderate explained variance [27].

## Author Contributions

Yang Zheng: conceptualisation, methodology, formal analysis, writing–original draft and writing–review and editing. Hongyan Yang: conceptualisation, methodology, investigation, data curation and writing–original draft. Jie Zhao: investigation, resources and validation. Baocui Zhang: data curation, software, formal analysis and visualisation. Jingjing Luo: investigation, resources and validation. Haiyan Wen: data curation, software and formal analysis. Chengcheng Sheng: writing–review and editing, validation and visualisation. Qian Lu: conceptualisation, project administration, supervision and writing–review and editing.

## Funding

This study was supported by National High Level Hospital Clinical Research Funding (Scientific Research Seed Fund of Peking University First Hospital) (Grant No. 2024SF73).

## Ethics Statement

This study was approved by the Biomedical Ethics Committee of Peking University. The ethics approval number for the study is IRB00001052‐24011.

## Conflicts of Interest

The authors declare no conflicts of interest.

## Data Availability

The data that support the findings of this study are available from the corresponding author upon reasonable request.
